# Accounting for Biomass Carbon Stock Change Due to Wildfire in Temperate Forest Landscapes in Australia

**DOI:** 10.1371/journal.pone.0107126

**Published:** 2014-09-10

**Authors:** Heather Keith, David B. Lindenmayer, Brendan G. Mackey, David Blair, Lauren Carter, Lachlan McBurney, Sachiko Okada, Tomoko Konishi-Nagano

**Affiliations:** 1 The Fenner School of Environment and Society, Australian National University, Building 48, Canberra, ACT, Australia; 2 Griffith Climate Change Response Program, Griffith University, Queensland, Australia; 3 Fujitsu Laboratories Ltd., Kawasaki, Japan; Montana State University, United States of America

## Abstract

Carbon stock change due to forest management and disturbance must be accounted for in UNFCCC national inventory reports and for signatories to the Kyoto Protocol. Impacts of disturbance on greenhouse gas (GHG) inventories are important for many countries with large forest estates prone to wildfires. Our objective was to measure changes in carbon stocks due to short-term combustion and to simulate longer-term carbon stock dynamics resulting from redistribution among biomass components following wildfire. We studied the impacts of a wildfire in 2009 that burnt temperate forest of tall, wet eucalypts in south-eastern Australia. Biomass combusted ranged from 40 to 58 tC ha^−1^, which represented 6–7% and 9–14% in low- and high-severity fire, respectively, of the pre-fire total biomass carbon stock. Pre-fire total stock ranged from 400 to 1040 tC ha^−1^ depending on forest age and disturbance history. An estimated 3.9 TgC was emitted from the 2009 fire within the forest region, representing 8.5% of total biomass carbon stock across the landscape. Carbon losses from combustion were large over hours to days during the wildfire, but from an ecosystem dynamics perspective, the proportion of total carbon stock combusted was relatively small. Furthermore, more than half the stock losses from combustion were derived from biomass components with short lifetimes. Most biomass remained on-site, although redistributed from living to dead components. Decomposition of these components and new regeneration constituted the greatest changes in carbon stocks over ensuing decades. A critical issue for carbon accounting policy arises because the timeframes of ecological processes of carbon stock change are longer than the periods for reporting GHG inventories for national emissions reductions targets. Carbon accounts should be comprehensive of all stock changes, but reporting against targets should be based on human-induced changes in carbon stocks to incentivise mitigation activities.

## Introduction

Stabilising the carbon stock in the atmosphere to prevent dangerous anthropogenic interference with the climate system is the objective of the United Nations Framework Convention on Climate Change (UNFCCC) [1]. Forest ecosystems naturally exchange carbon dioxide (CO_2_) with the atmosphere, but losses of carbon stocks from forests due to land management activities is a significant source of greenhouse gas (GHG) emissions [2]. Losses of carbon as emissions of CO_2_ from forest ecosystems to the atmosphere occur due to biological decomposition processes, natural disturbance events like fire, as well as human activities. Removals of CO_2_ from the atmosphere to forest ecosystem carbon stocks occur by the uptake of carbon in growing vegetation.

Under the UNFCCC and Kyoto Protocol, each country constructs GHG inventories and reports on their net annual emissions, that is, flows of CO_2_ and other GHGs, due to specific activities and sectors including Land Use, Land Use Change and Forestry. Flow-based inventories in the land sector, however, obscure important differences between ecosystems types and between the impacts of human activities and natural disturbances. A more comprehensive, stock-based approach to accounting can address these problems and provide information that complements flow-based accounts (Ajani et al. 2013). The flow-based accounts are currently used for national reporting against emission reduction targets over short time periods, such as annual or 5 to 8 year commitment periods, as determined by political negotiations. However, this short time period exposes nations to high variability in emissions due to natural disturbance events, especially wildfires, which are outside human control, yet create large GHG fluctuations. The resulting variability in national net emissions due to natural disturbance can be much greater than the changes due to land use impacts, thus confounding attempts to provide incentives for reducing emissions in the land sector through improved forest management.

Losses of carbon due to wildfire are a significant component of global annual GHG balances and so it is important that they are included in national inventories. Seasonal losses of carbon from wildfires are detected as high concentrations of trace gases in the troposphere [3], [4]. Estimates of gross losses of carbon from forest fires globally range from 2.0–2.5 Pg C yr^−1^ [5], [6] to 3.8–4.3 Pg C yr^−1^ [7] as annual averages. As a comparison of the magnitude of this flux, the global net losses are 1.1 PgC yr^−1^ from land-use change and 7.9 PgC yr^−1^ from fossil fuel emissions [8]. For nations with large areas of forest that are subject to periodic wildfires, such as Australia, Canada, USA, Russia and South Africa [9], [10], [11], the treatment of wildfires in carbon accounting for forest management is an important consideration. The rules and methodologies are complex, often controversial, and raise many issues in international accounting systems.

Carbon stock changes due to wildfires occur at different temporal scales that should be included in comprehensive stock-based accounts. Large losses occur over periods of hours to days or weeks due to combustion and are highly variable each year due to occurrence of fire. Uptake through forest regrowth and losses through decomposition of dead biomass occur over decades to centuries. These changes in carbon stocks are balanced in comprehensive accounts, and in the accumulated concentration of CO_2_ in the atmosphere, when assessed over a sufficiently long time period, assuming the fire regime is stationary. The following information is required to enable inclusion of wildfire disturbance in comprehensive stock-based national carbon accounts: carbon stock loss due to combustion, redistribution of carbon stocks between living and dead biomass components; subsequent rates of decomposition; and carbon uptake by regenerating vegetation. These estimates of carbon stock change need to be calculated on a landscape-wide basis and integrated over appropriate time periods [12] with data from a range of forest types. However, methodologies for measuring carbon stock change due to wildfire are problematic for a number of reasons, including: (i) wildfires typically extend over large areas, within which fire severity varies spatially; (ii) initial carbon stocks vary across the landscape in response to differences in forest type, age, disturbance history and environmental factors; (iii) biomass components are combusted with differing efficiencies; (iv) fire occurrence is stochastic [13], [14]; and (v) the dynamics of carbon stocks occur over long time periods in response to wildfire.

Of these methodological issues, the parameter with the greatest uncertainty is the amount of carbon combusted, calculated as either an amount or a proportion of the fuel load or total stock, and then upscaled from individual components to sites and landscapes [5], [15]. Combustion efficiency varies due to characteristics of individual fuel materials, such as logs of varying size, wood density, state of decay and moisture content [16], and characteristics of the fire, such as intensity and residence time, which determine the oxidation conditions of flaming or smouldering combustion [7], [17], [18]. At the landscape scale, the proportion combusted depends on the areas burnt within the fire boundary, including small scale heterogeneity in fire occurrence and severity. The high degree of uncertainty created by the limited existing data is demonstrated when aggregated spatially in regional and global inventories. For example, estimates of carbon stock losses from wildfires in Australia derived from global analyses differ by up to six-fold [6], [19], [20], [21], [22].

The objectives of this study were to investigate the magnitude and timeframe of carbon stock changes due to wildfire and to evaluate the results in terms of implications for national carbon accounts.

- We assessed the magnitude of carbon stock losses due to combustion in a wildfire in terms of the amount and proportion of the total forest ecosystem carbon stock. We then considered the significance of these carbon losses compared with potential impacts from human activities.
- We considered the impact of fire on the ecosystem carbon stock in terms of the timeframe of stock changes. The mean residence times of carbon stocks change in response to redistribution of carbon among biomass components post-fire and their subsequent relative rates of losses and gains. We investigated the dynamics of carbon stock change within the current disturbance regime and not predicted future regimes or climate.
- We evaluated our results about the magnitude and timeframe of carbon stock changes in terms of their significance for the rules and methodologies governing national carbon accounting for forest management. Now is an important time to contribute quantitative information to the development of accounting guidelines for post-2020 agreements which include forest management and disturbance events under the Durban Platform for Enhanced Action [23].

To address these issues about accounting for carbon stock changes due to wildfire, we examined changes in carbon stocks in a temperate eucalypt forest in south-east Australia that was subjected to a wildfire in 2009. We selected a temperate forest because they contain high carbon stocks and contribute 34% to the global forest carbon sink; a proportion that has been increasing over the last two decades [24]. Changes in carbon stocks due to combustion during the fire were assessed empirically and we also investigated the long-term dynamics of biomass carbon pools post-fire using a simulation model, both at the site level and up-scaled across the landscape.

## Methods

### Description of study region

**Figure 1:**
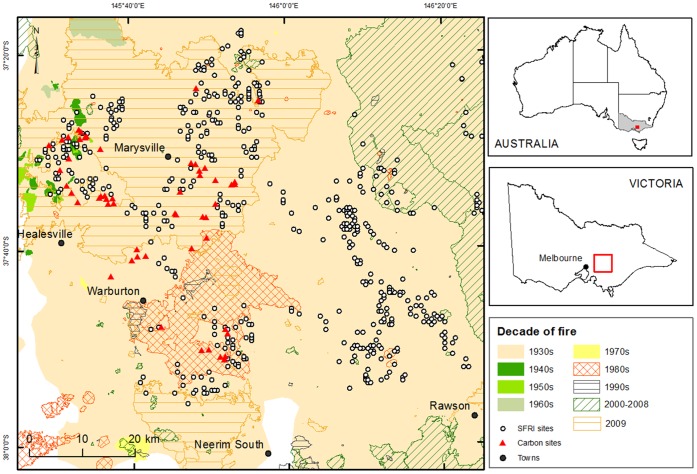
Location of the study region in the Central Highlands of Victoria, Australia. Location of field sites is marked, including long-term ecological monitoring sites (n = 54) where carbon stocks in biomass components were measured, and inventory sites (n = 876) where carbon stocks were estimated and used for upscaling. The spatial extent of wildfires shown represents the outer boundary of the burnt area.

Access to field sites was granted by Parks Victoria and the Department of Environment and Primary Industries Victoria. Our study region (2,326 km^2^) was the tall, wet sclerophyll montane ash forests (*Eucalyptus regnans, E. delegatensis* and *E. nitens)* in the Central Highlands of Victoria (Figure 1). These temperate forests are among the most carbon-dense in the world and protection of these carbon stocks offers the potential for mitigation [25]. These forests are subject to a disturbance regime of infrequent, high intensity wildfires [26], [27].

### Wildfire disturbance regime

The known historical fire frequency has been one in the 1700s, three in the 1800s, nine in the 1900s, and three in the 2000s [27], [28], and their spatial extent was mapped across the study region (Figure 1). Occurrence of these fires does not necessarily mean that each conflagration occurred at the same locations or at the same intensity. The mean interval for fires that kill ash trees at a site has been estimated to be 75 to 150 years [29], [30]. However, an estimate that approximately half the trees survive within the boundary of a wildfire [30] means that only every second fire in any one location is of sufficient intensity to kill ash trees. Hence, the mean interval of all fires is estimated to be 37 to 75 years. A maximum fire interval of 350–400 years was estimated based on maximum age of the montane eucalypt species [31].

Unlike most eucalypt species, *E. regnans* and *E. delegatensis* are obligate seeders and usually killed if their canopies are predominantly scorched by fire [32], [33], [34]. However, wildfires are highly variable in extent and severity, which is reflected in current stand age structures of a mosaic of even-aged and multi-aged forests [26], [35], [36]. Even-aged stands result from widespread mortality and regeneration following high-severity wildfire. Multi-aged stands result from partial tree death and subsequent regeneration alongside surviving trees [29], [37], [38]. Hence, the variability of historical fires is important for determining current stand structures. For example, our entire study area is within the boundary of the 1939 fire. However, evidence that individual trees and forest stands survived is seen by the current age structure of patches of old-growth forest where many living trees have fire scars from the 1939 fire event [39].

**Figure 2:**
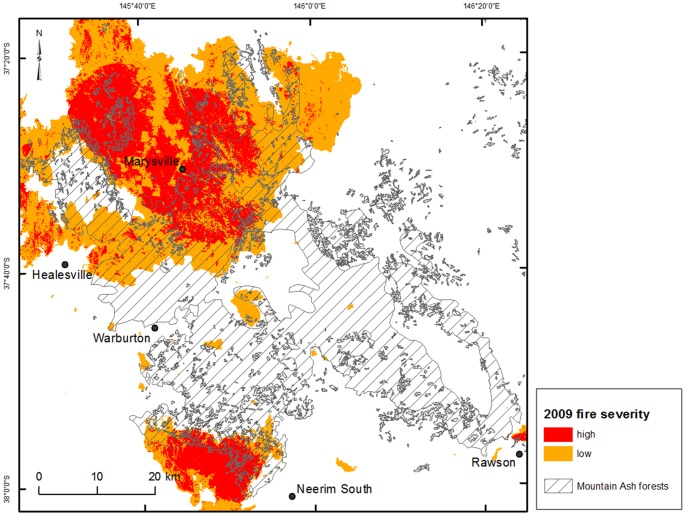
Spatial extent of the 2009 wildfire within the study region. The fire was categorised as low-severity where most trees survived, and high-severity where most trees were killed.

A wildfire started on 7^th^ February 2009 that burnt over 450,000 ha in Victoria under extreme hot, dry weather conditions (46.4 °C in the nearby city of Melbourne), with winds up to 100 km hr^−1^ [40], [41]. Within our study region of montane ash forest, 485 km^2^ burnt at low-severity and 283 km^2^ burnt at high-severity (Figure 2). Fire severity was mapped by the Victorian Department of Environment and Primary Industries using air photo interpretation and categorised by degree of crown scorch and subsequent tree survival or mortality.

### Field sites

We selected 54 sites within the montane ash forest region of the Central Highlands of Victoria (Figure 1) from an existing network of 170 long-term ecological monitoring sites [27] to provide sites with a range of conditions of forest age and severity of the 2009 wildfire. The site categories of forest age were 1983 and 1939 regrowth after wildfire followed by salvage logging or clearcut logging, and old growth. Salvage logging was common practice after wildfire to remove the timber resource [42], [43], [44], [45]. Selection of these regrowth sites provided reasonably even-aged stands of similar structure, although some residual older trees occur [46]. Old growth forest consisted of dominant trees greater than 120 years old but mostly 250 years old that had regenerated following a fire in approximately 1750 [39]. The site categories of severity of the 2009 wildfire were unburnt, low-severity and high-severity burns. Our distinction between low- and high-severity fire was based on site observations of full scorch of the canopy and mortality of *E. regnans and E. delegatensis* trees. The three forest age and three fire severity categories produced a matrix of nine categories, each with six replicate sites.

While the 54 sites covered a range of forest stand structures and disturbance histories, the assumption that the sites had comparable pre-fire biomass carbon stocks, disturbance history, and local biophysical conditions did not necessarily hold. Their carbon stocks likely varied because of the natural heterogeneity of the landscape and the fact that fires are less likely to burn in wet, highly productive mature and old growth forest areas [47], [48], [49]. Furthermore, the monitoring protocols used prior to the 2009 fire at the sites were aimed at biodiversity assessments, and did not include all the measurements required to quantify ecosystem carbon stocks. Therefore, we undertook additional field survey to ensure there was a consistent set of measurements across sites.

### Measurement of biomass carbon stocks and stock loss due to fire

**Figure 3:**
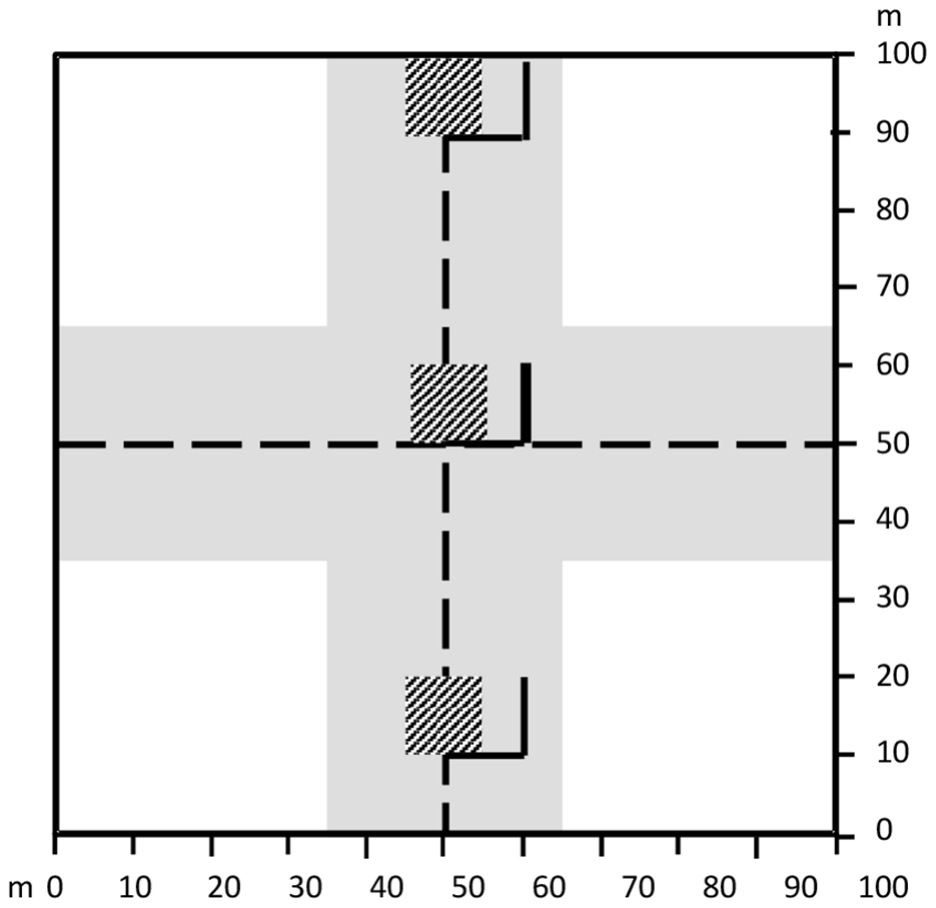
Design of 1 ha experimental sites. Sites of 100 m×100 m included central and perpendicular transects (dashed lines), 10 m×10 m plots (hatched), 10 m transects (black lines), and area sampled for large trees 2×30 m×100 m = 0.51 ha) (grey shaded area). Different biomass components were sampled in different parts of the site.

We measured carbon stocks in all aboveground biomass components in the forest (living vegetation, dead standing trees, coarse woody debris (CWD or dead and downed woody debris), and litter) at the selected 54 sites in 2010. We defined biomass as all intact organic components of the ecosystem, both living and dead, above- and below-ground, but excluding soil organic matter. Biomass components were classified for ease of measurement and to track movement of carbon between ecosystem components. Classification was based on the status of living or dead, size and vertical location. We used different sampling strategies for each biomass component within 1 ha sites to maximise accuracy and efficiency of measurements of different sizes, densities and distributions of components (Table 1 and Figure 3).

**Table 1:** Methods for measuring carbon stocks in biomass components within 1 ha sites post-fire at burnt and unburnt sites.

Biomass component	Sampling strategy	Measurement
**Trees (living and dead) eucalypt and rainforest species**		
<100 cm diameter	Three 10 m×10 m plots (0.03 ha)	Estimated height and diameter in size categories
>100 cm diameter	Two perpendicular intersecting 100 m×30 mtransects (0.51 ha)	Measured DBH and height.
**Mid- and Under-storey (living and dead) non-eucalypt species**		
<20 m height	Three 10 m×10 m plots (0.03 ha)	Estimated height and diameter in size categories
**Coarse woody debris**		
<60 cm diameter	Line intersect method along 6×10 m transects	Log diameter, decay class, hollows, charcoal and bulk density [64], [65]
>60 cm diameter	2×100 m transects	
**Litter layer**		
<2.5 cm diameter	15 points along transects per site. 30 quadrats randomlylocated at sites to cover range in litter depths	Measured litter depth.
		Measured litter dry mass.

All woody stems, both living and dead, greater than 2 m height and less than 100 cm diameter (diameter at breast height, DBH) were assessed in height and diameter categories for each species within three 10 m×10 m plots (0.03 ha). Trees greater than 100 cm DBH were measured in two perpendicular and intersecting 100 m transects by 30 m width (0.51 ha). In large trees that form buttresses with fluted stems [50], there is a cross-sectional area deficit that was accounted for by converting to a ‘functional’ diameter [51]. The allometric equation for *E. regnans* derived by Sillett *et al*. [51] was used to calculate stem and branch volume, and multiplied by wood density and carbon concentration to derive aboveground biomass carbon content. Average stem wood density was 0.520 g cm^−3^ [52], [53], [54], [55] and branch wood density was 0.677 g cm^−3^ [51]. A carbon concentration of 0.5 gC g^−1^ was used for all biomass components [56], [57]. Internal decay or hollows in stems were accounted for in the calculation of biomass derived from stem volume [58], [59]. Equations to predict occurrence and volume of decay related to tree size were derived from a subset of inventory data (734 trees) [60] where dimensions of defect in sawlogs had been measured. Allometric equations for other vegetation components included *Acacia* spp as mid-storey trees [61], a general rainforest equation for other mid- and under-storey species [62], and treeferns were calculated as a cylindrical volume and measured wood density. Root mass in *E. regnans* forests has not been measured, hence an average root: shoot ratio for eucalypt forests of 0.25 [63] was used to convert aboveground biomass to total tree biomass for living and dead trees.

Coarse woody debris consisted of all woody material ≥25 mm diameter on the ground, and was measured using the line intersect method [64], [65]. Logs less than 60 cm diameter were measured along 6×10 m transects within the site and logs greater than 60 cm diameter were measured along 2×100 m perpendicular transects. Each piece of CWD was assessed for hollows and degree of decay in three categories. Bulk density was measured for each category of decay to determine biomass. The litter layer consisted of organic material less than 25 mm diameter including leaves, twigs, insect detritus, animal scats, and comminuted material that was recognisable as organic material. Litter depth was measured at 15 points per site on a compressed litter pack to standardise the quantity of loose material. Depth was converted to biomass using a relationship derived from 30 quadrats sampled for dry weight of litter. Our results for carbon stocks refer to the total biomass of living and dead, above- and below-ground components.

**Figure 4:**
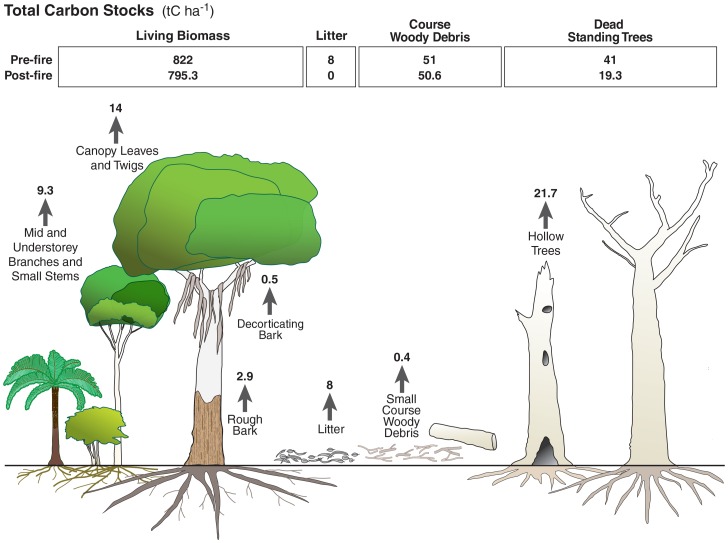
Schematic of carbon stocks in the forest ecosystem and stock changes resulting from fire.

We estimated biomass carbon stock loss by combustion in the 2009 wildfire from the following lines of evidence: (i) field observations and photographs taken immediately after the fire (within 2 months) [66], (ii) measurements of some components of the forest that were measured pre-fire compared with post-fire, and (iii) measurements of biomass components post-fire taken at burnt and unburnt sites. Biomass components combusted in the fire were measured specifically; including, hollow trees, decorticating and rough bark, canopy, shrub biomass, CWD and litter (Table 2 and Figure 4).

**Table 2:** Methods for measuring changes in carbon stocks of biomass components after wildfire, within 1 ha sites.

Biomasscomponent	Sampling time	Sampling strategy	Measurement	Proportion of biomass combusted	
				Low-severity fire	High-severity fire
Hollow trees	Measured in 2005and 2011	All trees in 1 ha	Measured DBH and height.	0.12 to 0.29	0.16 to 0.56
Decorticating bark	Post-fire, burntand unburnt sites	Representative sizerange of trees.	Measured relationship between treeDBH and bark mass.	1.0	1.0
Rough bark	Post-fire, burntand unburnt sites	Representative sizerange of trees.	Measured relationship between treeDBH and bark thickness plus bulk density.	0.3 (for trees>200 cm)	0.6 (for trees>200 cm)
Canopy leaves	Post-fire, burntand unburnt sites	Observations and photos	Estimated biomass from litterfall and leaf longevity	0.5	1.0 (mostly)
Canopy twigs	Post-fire, burntand unburnt sites	Observations and photos	Relationship between leaf and twig mass.	0.5	1.0 (mostly)
Shrubs	Post-fire,burnt sites	Representative range ofshrub sizes and species.	Minimum size of tips remaining onstems after fire. Calculated biomass in each stem diameter class in plots.	0.25 to 0.5 (frombranch diameter<5 cm)	0.3 to 0.75 (frombranch diameter<5 cm)
Coarse woodydebris	Post-fireburnt sites	Line intersect transects	Proportions of logs charred. Linesof charcoal remaining post-fire.	Highly variabledependent onlog size	Highly variabledependent onlog size
Litter	Photos soonafter the fire	Average from all low- andhigh-severity burnt sites.	Proportion of litter layer combusted.	1.0	1.0

Hollow trees, both living and dead, are highly susceptible to combustion because fire is funnelled through the pipe [67]. Data for individual tree heights and diameters from 2005 and 2011 [67] were compared to assess the change pre- and post-2009 fire. The difference in carbon stock between years represented a combination of natural mortality, decay and collapse of trees plus combustion at the burnt sites. Carbon stock loss was calculated as a proportion of the initial stock and the difference between burnt and unburnt used to distinguish the effect of natural decay and combustion.

Biomass of decorticating or ribbon bark hanging from the upper stem and branches was related to tree DBH by a non-destructive sampling method using estimated standard units [68]. All decorticating bark was combusted in the fire. Biomass of rough or fibrous bark on the lower stem was estimated from measurements of bark thickness and bulk density related to tree DBH. Combustion of rough bark was determined from the difference between burnt and unburnt sites. Both decorticating and rough bark mass were calculated for all the inventory trees at the 54 sites.

Canopy leaf biomass was estimated to be approximately 7 tC ha^−1^, derived from annual litterfall of 4.7 tC ha^−1^ yr^−1^ in ash forests [69], [70] and longevity in the canopy of about 18 months [50], [71]. Twig (diameter <4 mm) biomass is a similar amount to the leaves [72]. The proportion of the canopy combusted was estimated from photographs after the fire of the leaves remaining in the canopy and the scorched leaves that had fallen to the ground.

Combustion of shrub biomass in woody stems, branches and twigs was assessed using measurements of: (i) minimum sizes of tips remaining on stems after the fire, (ii) the proportion of biomass lost from a shrub when different tip sizes were removed, and (iii) the amount of biomass in each stem diameter size class of shrubs from the inventory data. A range of species and sizes of understorey shrubs was sampled.

Combustion of CWD was determined on each piece along the measurement transects by the proportion of the piece charred, or by the number of lines of charcoal remaining on the ground. The initial carbon stock of the logs that were completely converted to charcoal was assumed to be represented by the mean size of logs at the site. CWD was too variable between burnt and unburnt sites within stand age categories to allow a comparison to be indicative of the amount combusted as the mean of a category. The proportion of the litter layer combusted was estimated from photographs of quadrats taken soon after the fire in low- and high-severity burnt areas and compared with litter biomass in unburnt sites.

### Spatial estimation of carbon stocks

Spatial up-scaling of the site data to the landscape level followed the statistical modelling approach of Keith et al. [73]. Calibration data for the model used the pre-2009 carbon stocks from the 54 sites described above, as well as an additional 876 sites with inventory data [60]. The statistical model regressed site carbon stocks against the following explanatory variables at a 250 m grid resolution: climate variables of precipitation, water availability index, temperature (minimum, mean, maximum) and radiation; topographic variables of topographic position, elevation, aspect and slope; substrate variables of soil parent material, lithology and soil organic carbon content; gross primary productivity (GPP, derived from remote sensing); forest type; forest management area; and disturbance history. Methods used to derive spatial estimates of the environmental and GPP variables are documented in Mackey et al. and Berry et al. [26], [74], [75]. Disturbance history included wildfires and logging under different silvicultural systems, both of which have complex effects on forest age structure. The statistical regression model was used to generate spatial estimates of carbon stocks across the study region.

Impacts of the wildfire on ecosystem carbon stocks were quantified by determining first, the loss in carbon stock of each biomass component combusted, and second, the redistribution of carbon among biomass components within the ecosystem. Spatial estimation of the carbon stock post-2009 fire was based on measured reductions in the stock according to the fire severity category in the burnt areas and forest age.

### Modelling change in carbon stocks

Changes in carbon stocks were simulated for each forest age category after high-severity fire at the average historical return time of 112 years. Initial conditions for the model used site data of the current carbon stock in each age category and the proportion of the stock combusted. After the fire, carbon stocks were redistributed among living and dead components according to the proportions measured in each age category. For the second fire cycle, the initial condition of total biomass at the time of the fire was the stock simulated for year 112, and the proportions of the stock combusted and redistributed were taken as the average of the site data from the 1939 regrowth and old growth forests. Changes in carbon stocks over time were simulated using functions to describe the processes of regeneration, mortality, collapse of dead trees, and decomposition of CWD derived from existing data and observations for montane ash forest (Table 3).

**Table 3:** Input data and equations to predict biomass carbon stock (B in tC ha^−1^) as functions of time (t) since disturbance in the model of change in carbon stocks after a high-severity wildfire in old growth montane ash forest.

Process	Function	Data	Source
Regeneration( = gain in carbon)	B_regen_ (t) = 1200×(1–exp (−0.0045×t))^0.7^	Inventory data from 99 sites	46,60
	Log_10_(SD_t_) = 1.28+3.16×0.913^t^ +1.9×0.99^t^Log_10_ (SD_t_) = 11.61–1.624 log_10_ (t)(average from the two functions used)	Double exponential function fitted tochronosequence site data for *E.regnans.*Function derived from site data	76,77
Mortality of regeneration( = input to dead trees)	B_dead_(t) = [(SD(t –1) – SD(t))/SD(t)]×B_regen_(t)	Empirically derived from stem density changes	Current
Branch fall ( = input to CWD)	B_branch_(t) = B_regen_ (t)×0.005	Rate constant derived empirically toproduce CWD biomass in therange observed (3–255 tC ha^−1^).	96
Mortality of surviving trees( = input to dead trees)	B_dead_(t) = 135 exp (−0.015×t)	Rate of mortality estimated from site data.	67
Collapse of dead trees( = input to CWD)	B_CWD_in_(t) = 687/(1+ exp(0.1×t −5)	Logistic function derived to fit observations thatdead trees remain standing for 10 to 75 years.	66
Decomposition of CWD( = loss of carbon)	B_CWD_loss_(t) = 124 exp (−0.07×t)	Rate constant. Modelled range in CWD consistent withsite data (mean of 51 tC ha^−1^ and max. of 255 tC ha^−1^)	55,96 Current

The rate of regeneration was based on carbon stocks at sites with different times since stand-replacing disturbance, derived from the current study and inventory data [60]. Mortality due to self-thinning of the regeneration was estimated from changes in stem density with age, based on data from the literature [76], [77]. Mortality of living trees that survived the fire was based on measured rates of mortality over 28 years [67] and the average longevity of *E. regnans* of at least 250 years [78], but up to 500 years [79]. The rate of collapse of dead standing trees was described by a logistic function, with a slow initial rate that increased to a maximum and then a slow final rate when few trees remain. This pattern was based on observations and evidence that dead trees remain standing for approximately 10 to 75 years [66], [80]. The biomass in dead trees was then transferred to the CWD pool. The rate of decomposition of CWD and dead root biomass used in the model was 0.07 yr^−1^, which had been derived from empirical data for *E. regnans* [55]. This rate was intermediate among published rate constants [80], [81], [82], and produced amounts of CWD that were consistent with observed amounts in montane ash forest.

## Results

### Current carbon stock

**Figure 5:**
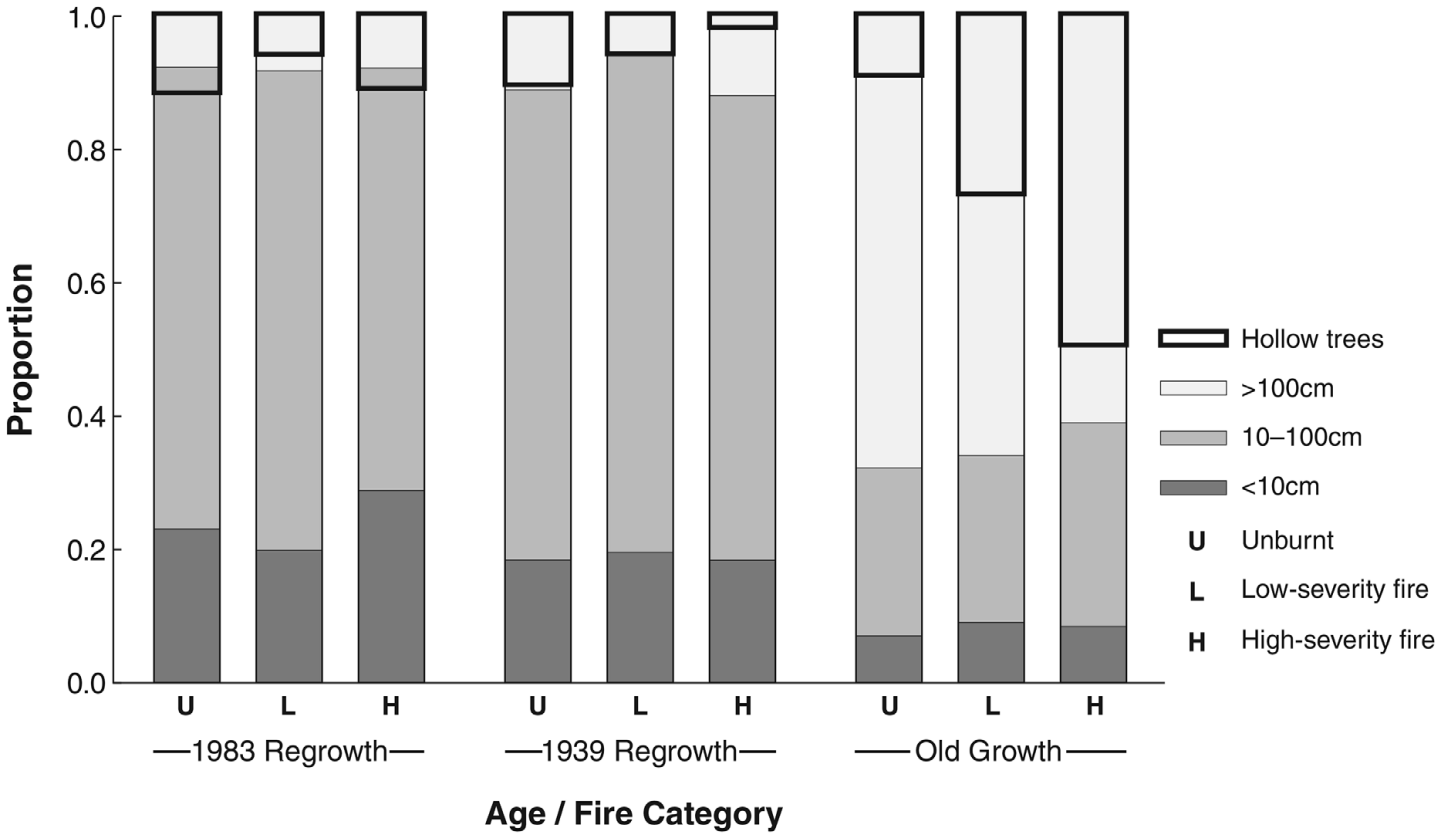
Distribution of carbon stocks in trees according to their size. Carbon stock is shown as the proportion in stem size classes of DHB and trees with hollows, averaged for the sites in each age/fire category (n = 6 sites per category).

Biomass density varied substantially among sites within the montane ash forest due to the disturbance history and subsequent age distribution of the forest. Biomass density (mean ±standard error, n = 6 sites) in unburnt stands was 405±33 tC ha^−1^ in 1983 regrowth, 603±74 tC ha^−1^ in 1939 regrowth, and 1039±44 tC ha^−1^ in old growth sites. The structure of these forest stands is illustrated in terms of the distribution of their carbon stock by tree sizes (Figure 5). These tree sizes demonstrate that the regrowth stands were not necessarily even-aged and included some larger residual trees which contained high, but variable, levels of biomass.

CWD biomass was highly variable among sites in the age/fire categories, ranging from 15 to 186 tC ha^−1^, with a mean of 67 tC ha^−1^. There was no significant difference in CWD biomass between forest age categories, reflecting the varied disturbance history of these forests where CWD remained after logging and wildfire, and its biomass was not related to the age of the stands. Litter biomass in the unburnt sites ranged from 6 tC ha^−1^ in the 1983 regrowth to 9 tC ha^−1^ in mature and old growth stands. This litter biomass corresponded to average site depth of litter when compressed of 2 cm and 3 cm, respectively, with the majority of measured points less than 5 cm and only a few points with 5–10 cm depth.

### Biomass components combusted

In areas burnt by low-severity fire, most of the over-storey trees survived as well as variable proportions of mid- and under-storey vegetation with green, scorched or combusted leaves. In areas burnt by high-severity fire, most of the leaves, twigs and decorticating bark in all canopy strata were combusted, although scorched leaves remained in patches. Some montane ash forests included a few *E. nitens* trees that regenerated by epicormic growth. Woody stems of most shrubs and mid-storey trees remained, but some were entirely combusted. The average branch size combusted from shrubs was 4 mm (maximum 19 mm) in low-severity fire, and 8 mm (maximum 42 mm) in high-severity fire.

The carbon stock losses from each biomass component and the proportion of the total biomass combusted differed with age class of the forest (Table 4). The distribution of readily combusted components within the ecosystem influenced the proportion of the stock emitted during the fire. Much of the observed change due to fire was combustion of shrubs in the understorey. However, this represented a small proportion of the total biomass in the ecosystem. Combustion of 7.8–16.2 tC ha^−1^ in the small branches on shrubs of all stem diameter categories in the understorey (Table 4) represented 1.6–4% of the total living biomass (above- and below-ground) in low-severity fire, and 1.9–6.7% in high-severity fire (range from young regrowth to old growth stands).

**Table 4:** Summary of biomass components combusted by low- and high-severity fires.

	Mass of carbon^1^ combusted (tC ha^−1^)					
	1983 regrowth		1939 regrowth		Old growth	
Low-severity fire	mean	SE	mean	SE	mean	SE
Litter	6	1.0	9	1.0	9	1.0
Canopy leaves	3.5		3.5		3.5	
Small stems	1.5	0.6	0.6	0.4	0.2	0.2
Small branches	11.9	1.2	11.8	2.7	7.6	3.4
Decorticating bark	0.3	0.02	0.3	0.06	0.5	0.07
Rough bark	0.6	0.2	0.6	0.1	1.3	0.3
CWD	1.4	0.6	1.8	0.7	0.2	0.2
Dead standing trees	3.8	0.9	9	1.7	17.6	5.2
Total biomass^2^ combusted^2^	29	4.5	36.6	6.7	39.9	10.4
Total carbon stock^3^	433	35	475	91	564	88
Proportion combusted^4^	0.063		0.072		0.066	
**High-severity fire**						
Litter	6	1.0	9	1.0	9	1.0
Canopy leaves	7		7		7	
Canopy twigs	7		7		7	
Small stems	2.2	0.5	0.8	0.3	0.3	0.2
Small branches	14	3.0	15	4.0	9	2.0
Decorticating bark	0.3	0.05	0.4	0.1	0.5	0.1
Rough bark	0.8	0.1	0.7	0.2	2.9	0.4
CWD	2.4	1.7	1.7	0.5	0.4	0.3
Dead standing trees	7	1.9	14.8	1.2	21.7	6.2
Total biomass^2^ combusted^2^	46.7	8.3	56.4	7.9	57.8	10.2
Total carbon stock^3^	28	46	459	102	589	63
Proportion combusted^4^	0.140		0.109		0.089	

1 Mass of carbon (mean ±standard error, n = 6 sites).

2 Total biomass combusted is the sum of all components combusted.

3 Total carbon stock (above- and below-ground) pre-fire is current carbon stock before the fire.

4 Proportion combusted is the total biomass combusted divided by the pre-fire carbon stock.

Some CWD was combusted during the fire, but both the absolute amount and proportion of initial biomass were highly variable spatially. Higher amounts of CWD were combusted in the 1983 and 1939 regrowth stands (range 1.37–2.41 tC ha^−1^) than in the old growth stands (0.16–0.43 tC ha^−1^) (Table 4). Large amounts of CWD occur in these regrowth stands due to self-thinning and residual material from previous logging and fire events. The high rates of combustion in regrowth stands occur because large proportions of the CWD are small logs and drier logs (decay class 1), compared with the old growth stands. Hollow or damaged trees, either living or dead, were a major source of carbon stock loss (3.8–21.7 tC ha^−1^) (Table 4). Where these trees had hollows in the centre of the stem, flames funnel up like a chimney and ignite exposed dry wood. In regrowth stands, hollow trees comprised 2–12% of the total tree biomass, and contained a carbon stock ranging from 7–50 tC ha^−1^. In old-growth stands, trees with hollows generally consisted of the older cohort of trees but also included damaged younger trees. They represented a highly variable proportion of the total biomass, from 9–49%, and contained a carbon stock of 45–241 tC ha^−1^.

The average carbon stock loss from biomass components was 40 tC ha^−1^ and 58 tC ha^−1^ in low- and high-severity fires, respectively, and this represented an estimated maximum amount (Table 4). As a proportion of the total biomass carbon stock (above- and below-ground), this loss represented 6–7% in low-severity fires and 9–14% in high-severity fires.

### Distribution of carbon among components due to fire

**Figure 6:**
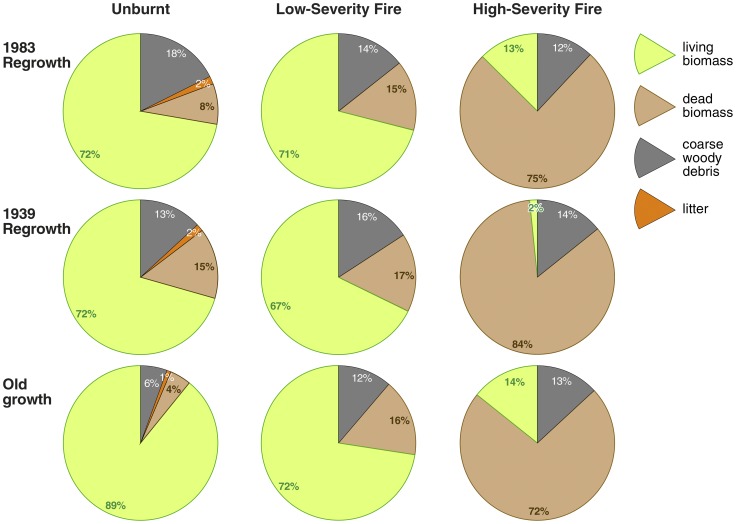
Distribution of carbon stocks among components in relation to stand age and fire severity. Distribution is shown as the proportion of carbon in each biomass component, including living vegetation, dead standing trees, litter and coarse woody debris.

The largest impact of fire on the ecosystem carbon stock was a shift in distribution among biomass components. The proportion of total biomass that was in living vegetation in unburnt sites was 72%, 71% and 89%, respectively in the 1983, 1939 regrowth and old growth sites. The shift from living to dead biomass was 3%, 6% and 19% due to low-severity fire, and 85%, 97% and 84% due to high-severity fire, respectively in the age categories (Figure 6). CWD increased by only a small proportion in the burnt sites; some logs were combusted and a similar amount was transferred into the CWD component.

### Carbon stock dynamics

**Figure 7:**
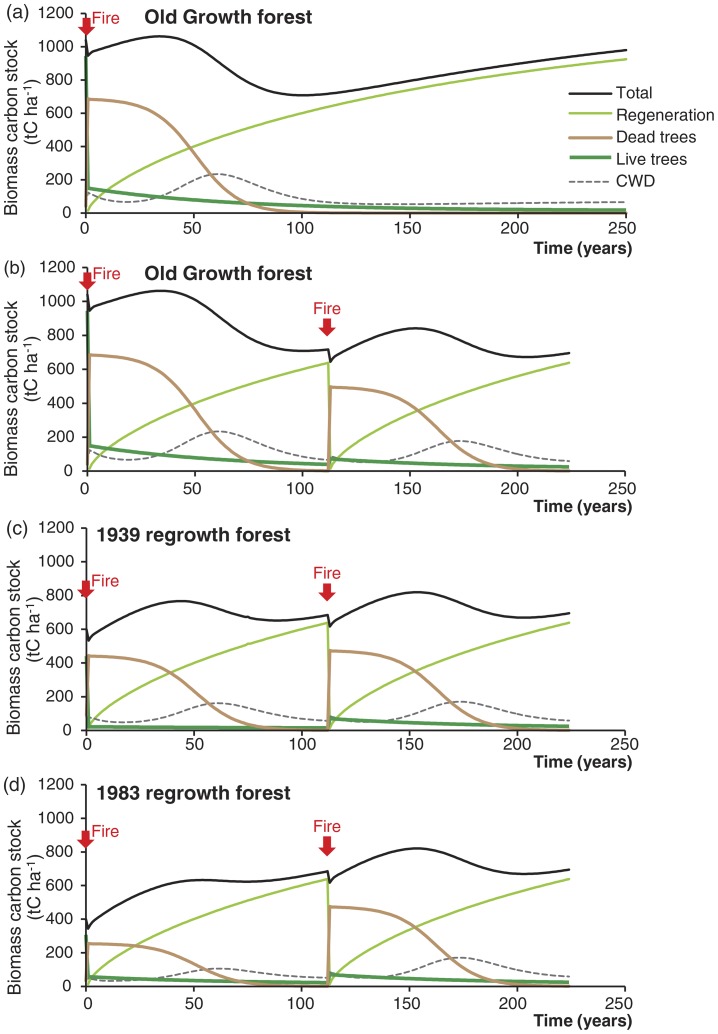
Simulated carbon stock change in biomass components of forests of different ages after high-severity wildfire. Initial fire occurred in year 1 and the change over time in each biomass component, plus the total biomass, is simulated; (a) old growth forest initially that is burnt in year 1 and recovery is simulated over 250 years assuming no other disturbance; (b) old growth forest initially that is burnt in years 1 and 113, according to the average fire frequency, and recovery simulated over two fire cycles; (c) 1939 regrowth forest initially that is simulated over two fire cycles; (d) 1983 regrowth forest initially that is simulated over two fire cycles.

Combustion of biomass during the fire resulted in an initial loss of some carbon, but most of the biomass remained at the site. Our simulation model predicted that carbon stocks would increase for several decades after the fire due to the combination of regeneration of living biomass and persistence of dead biomass components (Figure 7). Mortality of trees during the fire produced large amounts of dead biomass, but the trees only collapsed slowly and then contributed to the CWD pool, from where carbon stock loss by decomposition occurred over many decades. The lowest carbon stock in an old growth forest occurred nearly a century after a single fire when most of the dead biomass produced by the fire had decomposed. The maximum fluctuation in carbon stock over time as a result of fire was estimated to be 20% from the scenario of a single high-severity fire in old growth forest where there was no trend in total biomass due to changes in forest age (Figure 7a).

The carbon stock dynamics in the forests of initially different ages reflect the differences in the total biomass stock, its distribution among living and dead trees and CWD, the proportion combusted, and the proportions remaining in each of the components after the fire. The old growth forest had the largest increase in total biomass with the combined regeneration plus remaining dead trees, but subsequently the largest decline in total biomass as the dead trees collapsed and decomposed (Figure 7b). In the regrowth forests from 1939 and 1983, there was less dead biomass after the fire and the regeneration resulted in increasing total biomass carbon stock over most of the century following the fire (Figure 7c and d).

Under a regime of repeated fires on a 112-year return time, trends occurred in the total biomass carbon stock because forest age changed from that of the initial forest. The old growth forest decreased in carbon stock because of the more frequent disturbance, whereas the 1983 and 1939 regrowth forests increased in carbon stock because they remained undisturbed for a longer period than at their initial age. These scenarios of carbon stock dynamics represent an average forest stand of a given initial age subject to the average wildfire regime. A range of forest age classes occur across the landscape, including forests much older than 112 years. Forests that remain undisturbed for several hundred years have the capacity to continue accumulating carbon stocks.

### Spatial estimation of carbon stocks post-2009 wildfire

**Figure 8:**
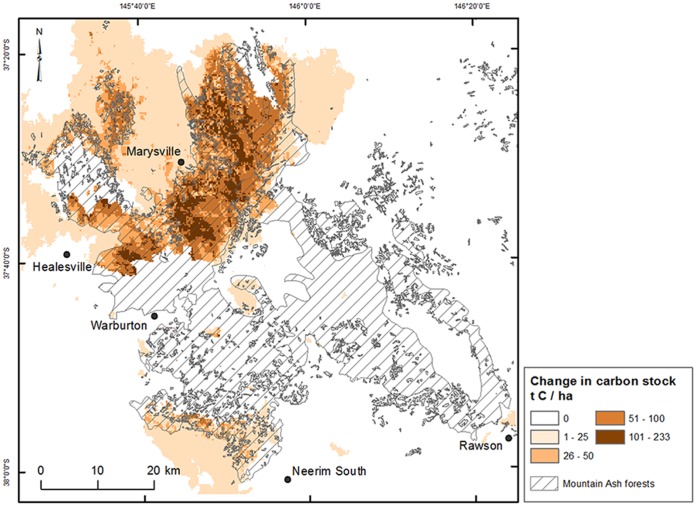
Spatially modelled change in carbon density (tC ha^−1^) across the montane ash forest region. Change in carbon stock was calculated as the difference between pre- and post-2009 wildfire, including total biomass of living and dead trees, above- and below-ground, shrubs, litter and coarse woody debris.

The carbon stock loss post-2009 fire was estimated spatially from the distribution of biomass carbon density in the unburnt montane ash forest and the proportion of biomass combusted in low- and high-severity fire for each forest age category (Figure 8). The total carbon stock in the montane ash forest region (2,326 km^2^) was 112.8 TgC pre-2009 and an estimated 3.9 TgC was emitted from biomass combustion during the 2009 wildfire (burnt area of 768 km^2^ or 33% of the area of montane ash forest). The carbon emitted represented 8.5% of the total biomass carbon stock in the area of montane ash forest that was burnt.

## Discussion

Although the total amount of carbon stock loss across the landscape burnt during the wildfire was large as an annual flow in the national inventory, we found that the proportion of total ecosystem carbon stock combusted was relatively small, both at site and landscape scales. The major impact of wildfire was the redistribution of carbon among biomass components, from living to dead biomass and CWD, and their longer-term rates of change due to decomposition and regeneration. Assessing the carbon stock loss by combustion in relation to the total stock, rather than fuel load, meant that changes in stock could be tracked over time and compared between different human activities and disturbance types.

### Amount and proportion of forest ecosystem carbon stock combusted

We found that the proportion of total ecosystem biomass carbon stock combusted during a wildfire in the montane ash forest was 6–14% at the site scale depending on fire severity and forest age (27 years to old growth), with an average of 8.5% across the landscape that was burnt. This is a relatively small proportion compared with impacts of human disturbance events, such as logging that removes biomass off-site [46], [83], [84]. Our results are similar to studies in other forest ecosystems (boreal, temperate mixed conifer, savannah woodlands, and other temperate wet sclerophyll) that estimated the relationship between carbon stock loss with total stock, but are lower than estimates of proportion of biomass combusted related to fine and coarse fuel loads (data and references in Table S1).

The estimated loss of only a small proportion of ecosystem carbon stock combusted in a fire concords with simulation results of net losses of carbon by 4 to 8% over 180 years under a fire regime scenario of increased frequency and intensity in south-east Australia [85]. Under this scenario, the loss was due to increased mortality leading to a younger age distribution of trees. Similarly, a sensitivity analysis of the carbon balance model for Canadian forests showed that a three-fold increase in area burned decreased the total forest carbon stock by 6% over 100 years [86]. These predicted losses of carbon stocks are relatively small over a century or two compared with the impact of many human activities.

Differences in the proportion of fine fuel combusted in different ecosystems are insignificant in terms of the total ecosystem carbon stock, representing 0.5 to 1% or 0.5 to 3 tC ha^−1^. The proportion of coarse fuel combusted varies greatly depending on the size of material, which is influenced not only by forest age but also by disturbance history. Although proportions of biomass combusted were lower in montane ash forest than in other forest types, the amounts combusted in these fuel size classes were greater (Table S1). Amounts of carbon emitted during combustion in different ecosystems depend on vegetation type, past disturbances resulting in dead biomass, and fire weather conditions [87].

We conclude from our methodology that estimating carbon stock loss as a proportion of the total ecosystem carbon stock (based on tracking each biomass component) is a useful metric to compare with other impacts on ecosystem carbon stocks and other sectors of the national GHG inventory. This metric is thus more appropriate for national accounts than the current default reporting of emissions as the product of a combustion factor and fuel load in the IPCC guidelines and Australian national inventory report [6], [15], [82], [88], [89]. In our method, all biomass components are assessed without making assumptions about which components constitute fuel.

Our values of carbon stock loss in a wildfire may be small overestimates because some of the biomass combusted is not volatilised and therefore not emitted to the atmosphere. Under conditions of reduced oxygen, partial combustion forms char that remains on the ground, or particulate carbon that is transported in smoke but settles to the ground elsewhere. The proportion of char produced in wildfires has been estimated to be 1–10% of the biomass combusted [90]. Both these forms of carbon are highly resistant to decay and form a long-term stable component of soil carbon stocks [90].

### Changes in forest biomass carbon dynamics

The major impact of wildfire in the montane ash forest was the redistribution of carbon among biomass components, from living to dead, and subsequent long-term stock losses from decomposition and gains by regeneration. The relative rates of these processes and subsequent mean residence times of the biomass components determine the long-term dynamics of carbon stock in the ecosystem. These long-term dynamics resulted in changes of up to 20% about the mean stock, whereas direct losses from combustion represented 6–14% of the total biomass stock.

Mean residence time of the biomass components combusted is critical for determining the impact of wildfire on longer-term carbon dynamics. More than half the stock losses from combustion were derived from biomass components with short lifetimes; in the canopy, bark, litter layer and shrubs. These components are described as fine fuel, and this amount remains reasonably constant after about 10 years post-fire in a range of forest types [91]. For example, fine litter in montane ash forest has a mean residence time of 1–4 years [50], [70], and 85–95% of the litter mass has accumulated within a decade after fire [91]. Recovery of the carbon stock within a decade in the montane ash forest is within the range of up to 10–20 years for recovery of rates of carbon uptake estimated from eddy covariance measurements of net ecosystem carbon exchange at chronosequence sites across a range of forest types in North America [92].

The components combusted with longer mean residence times, such as CWD and large hollow trees, have variable stocks and proportions combusted depending on forest type, conditions for decomposition, logging and fire history. This coarse material contributed 18 to 45% of the carbon combusted in the montane ash forest, with an increasing proportion in older stands. The residence time of this coarse material is in the order of decades to centuries [67], [93].

Rates of carbon accumulation in regenerating stands are difficult to estimate due to limited site data of known forest age, particularly in older aged stands. We compared carbon uptake in regenerated living biomass at a given age estimated from our site data, for example, 3.8 tC ha^−1^ yr^−1^ at 80 years, with the net ecosystem exchange (NEE) estimated from eddy covariance measurements. An average NEE over 10 years of measurements was 5.8 tC ha^−1^ yr^−1^ and the maximum was 9.3 tC ha^−1^ yr^−1^ in an 80 year old stand of *Eucalyptus delegatensis* or Alpine Ash [94]. This average NEE is comparable to the 6 tC ha^−1^ yr^−1^ in a temperate forest in Oregon, which is the highest value reported for forests of a similar age in North America [92]. Carbon accumulated in living biomass does not include uptake of carbon that is then transferred to dead biomass pools and soil organic matter.

Our simulated carbon stocks for each biomass component were within the range observed in the field. Actual magnitudes of stocks at different ages were difficult to verify because of the confounding disturbances of wildfire and salvage logging that have occurred in these forests. Among our sites, there were no younger stands that had been burnt but not logged and such stands would be rare in the landscape. Our simulated turnover times appeared to be slower than in some other ecosystems, such as boreal forests [95]. However, observations of large logs remaining after 60–90 years in the montane ash forest [50], [96] suggested that even slower rates may be characteristic of large biomass components. Uncertainty about the magnitude of the changes and their timing in the long-term dynamics of carbon occurred mainly because of limited information about rates of tree fall and decomposition.

### Inclusion of wildfire in national carbon accounts

Changes in carbon stocks due to natural disturbances, such as wildfire, must be included in national carbon accounts. The current UNFCCC carbon accounting guidelines for forest management (Article 3 paragraph 4 clause 33) address carbon stock change due to natural disturbances under the ‘*force majeure*’ provision (Decision 2/CMP.7, CoP 17 Durban Climate Conference) [97]. Natural disturbances, such as wildfire, outbreaks of pests and diseases, extreme weather events and geological disturbances, are considered as extraordinary events that are outside human control. An average net stock change due to natural disturbances is calculated as a background level within the forest management reference level. These accounts for forest management and natural disturbances must be included in the 2015 inventory reports from signatory nations to the Kyoto Protocol (38 of the 43 Annex 1 nations), which are assessed for compliance against emissions reduction targets [98].

The current negotiations under the Durban Platform for Enhanced Action [23] represent a critical time to provide quantitative data on carbon stock changes due to disturbance and to interpret the implications of the inventory for maximising outcomes of mitigation activities. Nations can develop and choose methodologies, within the UNFCCC guidelines, that are appropriate for their accounts [99]. The default method is calculated as an average of net stock change from natural disturbances from 1990 to 2009, plus a margin of twice the standard deviation. Annual stock changes that are above this background level are excluded from the accounts. When this exclusion occurs, the area of land affected is removed from the inventory so that subsequent uptake by the regenerating vegetation is not counted, until pre-disturbance carbon stocks are restored [99]. The UNFCCC guidelines provide some flexibility, but the methodology should adhere to the objectives of the *force majeure* provision of minimising annual variability in the reported net stock changes due to natural disturbance events. The rationale is that this variability should not negate reductions in emissions due to human mitigation activities over the timeframe of the reporting [97].

Our results about the magnitude and timeframes of carbon stock changes due to wildfires suggest that carbon accounts need to be comprehensive and include all stocks and flows. Such accounts enable differentiation of stock changes due to natural disturbances and human activities. There are four reasons why constructing carbon accounts which differentiate carbon stock changes are beneficial.

First, our results show that in terms of national annual net change in carbon stocks, the magnitude of carbon stock loss due to combustion in a wildfire is sufficient to mask reduced emissions due to mitigation activities. Our estimated carbon stock loss was 3.9 TgC from a single (albeit large) fire in 2009 within the montane ash forest. The annual average for all fires in Australia is estimated to be 8.7 TgC yr^−1^ [100]. These single and average wildfire emissions represent about 27% and 59%, respectively, of Australia’s annual emissions reduction target of 14.7 TgC yr^−1^ [100]. Similar magnitudes of emissions from individual wildfires have been quantified elsewhere, for example 2.9 TgC from the 2003 Californian fires [101]. Hence, differentiation of carbon stock changes due to natural disturbances and human activities is important when national GHG inventory reports are used for assessing progress in meeting emission reduction targets over short time periods.

Second, we demonstrated that the changes in carbon stocks in response to a disturbance event occur over the following decades. Hence, derivation of a background level of net carbon stocks over a two decade period (1990–2009) is not adequate to account for the variations in carbon dynamics within a disturbed area. However, the carbon stock at the landscape scale represents the sum of forest stands that have been burnt at different times and intensities, thus producing a mosaic of stand age classes, amounts of debris, and stages of recovery from disturbance. Under a stable disturbance regime, the carbon stock at the landscape scale remains stable when accounted for over sufficiently long time periods. Hence, carbon stock losses from combustion of biomass components do not increase the accumulated atmospheric CO_2_ concentration under a stable fire regime. Carbon stock dynamics may alter at the landscape level if fire regimes change due to climate change, or due to the effects of growth from a forest age class distribution affected by historical activities [9], [85], [86], [102], [103], [104].

Third, the increases and decreases in carbon stock post-fire resulting from differential rates of regeneration, mortality and decomposition mean that excluding burnt areas from the national inventory until carbon stocks are restored to pre-fire levels, is not an adequate method for excluding the effect of natural disturbances on net changes in carbon stocks. In fact, the increase in carbon stocks for some decades post-fire would produce artificial credits, but later decreases due to decomposition would produce artificial debits in the national inventory, which are not related to human activities. Additionally, the high levels of uncertainty in these long-term dynamics could produce errors in the national inventory.

Fourth, an averaging period of net stock changes from a disturbance regime over two decades, or within the time period of adequate records, does not encompass the temporal scale of natural variability in wildfire regimes in many temperate forest ecosystems [105], such as montane ash forest with a return interval of many decades [30], [31]. The frequency and intensity of wildfire is influenced by interactions of large-scale features of atmospheric circulation, such as the El Niña-Southern Oscillation, Indian Ocean Dipole, Southern Annular Mode and the Sub-Tropical Ridge, which drive multi-decadal climate variability [13], [106], [107]. If a background level is to be used in an accounting system, then the preferred method would be to re-calculate the actual incidence of disturbance events at the end of the reporting period [108], [109], [110].

### Methodological and data uncertainties

Our results suggest that data from ecological monitoring sites can be used *a posteriori* to estimate carbon stock losses from wildfire, when supplemented with additional field data and ancillary data sources. Our approach used the basic framework of estimating carbon stock changes due to wildfire from the area burned, fuel load and combustion efficiency [87], [90] However, our sampling also incorporated spatial variability in fire severity and initial carbon stocks at the landscape level. This provided a more representative range in carbon stock losses at the regional scale. Covering this range is important even though a balanced experimental design is difficult to obtain in studies of stochastic disturbance events where field sites may not be comparable because of uncertainties in disturbance histories and stand ages. Previous studies in Australia have used either experimental fires imposed on a planned design of sites and pre-fire measurements, where the fires were either lower severity than wildfire or regeneration burns of logging slash [89], [111], [112], [113], or post-fire comparisons of burnt and unburnt sites albeit with limited replicate sites [41], [114].

Improved assessment of fire severity as a continuous variable at the landscape scale is important to allow more accurate estimates of carbon stock changes spatially [115]. New techniques in remote sensing are providing quantitative information about fire radiative power that can be related to factors that control fire severity [116], [117], [118]. However, adequate field calibration of the remotely sensed metric currently limits their application [116].

The results of up-scaling the site data to estimate carbon stocks and stock losses across our study region revealed that the effect of disturbance history on tree age structure and CWD was the major source of uncertainty in spatial estimates of biomass carbon stocks. Other studies have drawn similar conclusions that the determination of forest stand age is the main factor contributing to uncertainty in assessment of regional carbon stocks [5].

Our results suggest that current national accounting that uses default values (Tier 2 methodology) for fuel combustion efficiency and fuel load can be improved by using site data from different ecosystems. Data from existing state forest inventories need to be augmented by comprehensive sampling of forest age structure, including old growth forests, and all components of the carbon stock, including dead wood. The methods used here are generic and would constitute Tier 3 methodology that is appropriate for national and project-based accounting. The sampling methods were non-destructive and feasible to apply to a large number of sites.

## Conclusions

Carbon stocks in forest ecosystems change in response to wildfire in the short-term by combustion and in the long-term by redistribution among biomass components and their subsequent differential rates of decomposition and regeneration. It is important that carbon accounts include these processes because all stock changes, activities and land areas contribute to quantifying the global carbon cycle. However, the timeframes of the ecological processes that determine carbon stock changes are longer than the time periods for reporting of national inventories for compliance with emissions reductions targets. This problem of incongruous timeframes means that reporting against targets should be based on net changes in carbon stocks from human activities to incentivise mitigation activities. If the carbon accounts differentiate stock changes between the sources from natural and human-induced disturbances, then the net stock change from human activities can be extracted from the accounts for use in reporting. This distinction is currently made between the comprehensive UNFCCC accounts and the reports against targets for the Kyoto Protocol, except that the guidelines for the Protocol include net stock changes from natural disturbances that can be discounted above an averaged background level. However, our results indicate that the spatial and temporal variability in carbon dynamics is too high and the uncertainty of estimates too great to enable a realistic averaged background level that would prevent distortions of the inventory by stock changes that were outside human control.

## Supporting Information

Table S1 **Biomass combusted in wildfire from different forest ecosystems.**
(DOCX)
