# Supplementary material for: Accounting for Biomass Carbon Stock Change Due to Wildfire in Temperate Forest Landscapes in Australia
Source: PLoS One. 2014 Sep 10;9(9):e107126. doi: 10.1371/journal.pone.0107126 (PMC4160232; doi:10.1371/journal.pone.0107126)
Supplement: Table S1 — Biomass combusted in wildfire from different forest ecosystems. (DOCX) [file pone.0107126.s001.docx]

# Supporting Information

## Table S1. Biomass combusted in wildfire from different forest ecosystems

Comparison of biomass components combusted as an amount (tC ha^-1^) of carbon stock loss and as a proportion (%) of the stock in different biomass components from a range of forest ecosystem types.

| **Ecosystem** | **Fire type** | **Proportion** | **Amount (tC ha^-1^)** | **Source** |
| --- | --- | --- | --- | --- |
| Temperate wet sclerophyll eucalypt | Low- to high- | 6 – 14% of total carbon stock | 50 (29 – 58 range) | Current |
| forest, Victoria, Australia | severity wildfire | 75% of fine fuel, 20% of coarse fuel |  |  |
| Temperate *E. delegatensis* forest in | Experimental high- | 85 – 100% of fine fuel | 24 | 1 |
| SE Australia | severity fire | 26-70% of coarse fuel |  |  |
| Temperate wet, sclerophyll forest in southern Australia | Wildfire |  | 10 (ground fuel only) | 2 |
| Default value in Australian National Carbon Accounting System | Wildfire | 50% of fuel load |  | 3 |
| Savanna woodlands, northern | Experimental high- | 85 – 100% of fine fuel |  | 4 |
| Australia | severity fire | 32 – 39% of coarse fuel |  |  |
| Savanna woodlands, southern Africa | Experimental high- | 85 – 100% of fine fuel |  | 5,6 |
|  | severity fire | 32 – 39% of coarse fuel |  |  |
| Temperate wet, sclerohyll forest in Tasmania, WA and NSW, Australia | Prescribed fire | 45% (9 – 90% range) of coarse fuel |  | 7 |
| Temperate tall, open eucalypt forest in Victoria | Wildfire | 90% of coarse fuel (logs mostly < 50cm diameter) |  | 8 |
| Temperate mixed conifer forest, Oregon, USA | Low- to high-severity wildfire | 8.9 – 13.6% of total carbon stock | 29 | 9 |
| Temperate forest in the Pacific Northwest, USA | Wildfire | 22% | 25.5 | 10 |
| Boreal forest, Canada | Wildfire |  | 13 (biomass) | 11 |
|  |  |  | 3–24 (organic layer) | 12 |
| Boreal forest, Alaska | Wildfire |  | 20 (9-37 biomass) | 13 |
|  |  |  | 17 – 30 (biomass and organic layer) | 14 |

## References

1. O’Loughlin EM, Cheney NP, Burns J (1982) The Bushrangers experiment: Hydrological response of a eucalypt catchment*.* Proc First Nat Symp Forest Hydrology, Melbourne.

2. Cruz MG, Sullivan AL, Gould JS, Sims NC, Bannister AJ, Hollis JJ, Hurley RJ (2012) Anatomy of a catastrophic wildfire: The Black Saturday Kilmore East fire in Victoria, Australia. For Ecol Manag 284: 269 – 285.

3. Gould JS and Cheney NP (2007) Fire management in Australian forests. In: Raison RJ and Squire RO, editors. Forest management in Australia: implications for carbon budgets. National Carbon Accounting System Technical Report No. 32, pp 341 – 371.

4. Russell-Smith J, Murphy BP, Meyer CP (2009) Improving estimates of savannah burning emissions for greenhouse accounting in northern Australia: limitations, challenges, applications. International J Wildl Fire 18: 1-18.

5. Lindesay JA, Andreae MO, Goldammer JG et al. (1996) International Geosphere – Biosphere Programme / International Global Atmospheric Chemistry SAFARI-92 field experiment: background and overview. JGeophys Res 101: D19: 23521–23530.

6. Shea RW, Shea BW, Kauffman JB, Ward DE, Haskins CI, Scholes MC (1996) Fuel biomass and combustion factors associated with fires in savannah ecosystems of South Africa and Zambia. J Geophys Res 101: D19: 23551 – 23568.

7. Hollis JJ, Matthews S, Ottmar RD et al. (2010) Testing woody fuel consumption models for application in Australian southern eucalypt forest fires. For Ecol Manag 260: 948–964.

8. Hollis JJ, Anderson WR, McCaw WL, Cruz MG, Burrows ND, Ward B, Tolhurst KG and Gould JS (2011) The effect of fireline intensity on woody fuel consumption in southern Australian eucalypt forest fires. Aust For 74: 81 – 96.

9. Campbell J, Donato D, Azuma D, Law B (2007) Pyrogenis carbon emissions from a large wildfire in Oregon, United States*.* J Geophys Res 122: G04014.

10. Meigs GW, Donato DC, Campbell JL, Martin JG, Law BE (2009) Forest Fire Impacts on Carbon Uptake, Storage, and Emission: The Role of Burn Severity in the Eastern Cascades, Oregon. Ecosystems 12: 1246–1267

11. Amiro BD, Todd JB, Wotton BM et al. (2001) Direct carbon emissions from Canadian forest fires, 1959 – 1999. Can J For Res 31: 512–525.

12. de Groot WJ, Pritchard JM, Lynham TJ (2009) Forest floor fuel consumption and carbon emissions in Canadian boreal forest fires. Can J For Res 39: 367 – 382.

13. French NHF, Kasischke ES, Williams DG (2003) Variability in the emission of carbon-based trace gases from wildfire in the Alaskan boreal forest. J Geophys Res 108: doi:10.1029/2001JD000480.

14. Kasischke E S and Hoy EE (2012) Controls on carbon consumption during Alaskan wildland fires*.* Glob Change Biol 18: 685–699.
